# Band Gap Narrowing and Widening of ZnO Nanostructures and Doped Materials

**DOI:** 10.1186/s11671-015-1034-9

**Published:** 2015-08-29

**Authors:** Norlida Kamarulzaman, Muhd Firdaus Kasim, Roshidah Rusdi

**Affiliations:** Centre for Nanomaterials Research, Institute of Science, Universiti Teknologi MARA, Level 3, Block C, 40450 Shah Alam, Selangor Malaysia; School of Physics and Materials Studies, Faculty of Applied Sciences, Universiti Teknologi MARA, 40450 Shah Alam, Malaysia

**Keywords:** ZnO, Nanorods, Nanomaterials, Microstructure, Doping, Band gap, Narrowing, Widening, Energy level

## Abstract

Band gap change in doped ZnO is an observed phenomenon that is very interesting from the fundamental point of view. This work is focused on the preparation of pure and single phase nanostructured ZnO and Cu as well as Mn-doped ZnO for the purpose of understanding the mechanisms of band gap narrowing in the materials. ZnO, Zn_0.99_Cu_0.01_O and Zn_0.99_Mn_0.01_O materials were prepared using a wet chemistry method, and X-ray diffraction (XRD) results showed that all samples were pure and single phase. UV-visible spectroscopy showed that materials in the nanostructured state exhibit band gap widening with respect to their micron state while for the doped compounds exhibited band gap narrowing both in the nano and micron states with respect to the pure ZnO materials. The degree of band gap change was dependent on the doped elements and crystallite size. X-ray photoelectron spectroscopy (XPS) revealed that there were shifts in the valence bands. From both UV-visible and XPS spectroscopy, it was found that the mechanism for band gap narrowing was due to the shifting of the valance band maximum and conduction band minimum of the materials. The mechanisms were different for different samples depending on the type of dopant and dimensional length scales of the crystallites.

## Background

Zinc oxide is one of the most important metal oxides due to its unique physical characteristics of wide and direct band gap (~3.37 eV) with a large exciton binding energy (60 meV). It has attracted much attention among researchers and is used in various applications such as in optoelectronics [1–6], sensors [3, 7], pharmaceuticals [8], etc. It has been found that the band gap of ZnO can be changed by either decreasing the size of the crystallites [9–12] or by substitutional doping [13–15]. Substitutional doping can be done by a chemical reaction that forces the dopant element to be introduced into the crystal structure of the compound [13, 14]. Pure and single phase materials that are isostructural to the initial compound will be produced. These new compounds will exhibit novel characteristics that will be useful for future applications.

Although much work have been published on ZnO and ZnO-doped materials [1, 2, 9–15], there are still many unresolved issues on this subject. Band gap, as is well known, is a very important physical characteristic of materials affecting their electrical behaviour. Band gap change in ZnO nanostructures and doped ZnO is a phenomenon that is still not very well understood. This work is an ongoing effort to understand the basic fundamental reasons behind the observed band gap change phenomenon. The mechanisms, causes and reasons are investigated from the point of view of crystal structure, morphology, size and valence bands of the materials. As an example, there are no clear explanations of the mechanisms of band gap widening in nanostructured ZnO beyond the presence of vacancies as proposed by some researchers [16, 17] but without experimental proofs given of the existence of the vacancies. This work is focused on the explanation of the changes in band gaps of ZnO and doped ZnO materials with the support of experimental proofs.

Producing nanomaterials is not an easy task [7, 18]. Synthesis conditions have to be optimized in order to obtain pure, single phase nanostructured materials. The synthesis routes and parameters play an important role for obtaining certain types of morphologies and dimensions. Many synthesis routes and preparation methods give very little end products [6, 7, 19], and some synthesis process requires expensive equipment [20, 21]. Other synthesis methods used catalysts to obtain the nanostructures, and this affects the purity of the ZnO [1]. The method presented here is a simple wet chemistry method that has the advantage of producing large amounts of pure phase nanomaterials.

## Experimental

The synthesis of ZnO, Zn_0.99_Cu_0.01_O and Zn_0.99_Mn_0.01_O materials were prepared by using a simple sol-gel method. The starting materials used were zinc acetate dehydrate (R & M chemicals, 99.5 % purity), copper (II) acetate monohydrate (Riedel-de Haen, 99 % purity) and manganese (II) acetate tetrahydrate (Aldrich, 99 % purity). These materials were first dissolved in absolute ethanol and stirred for about 1 h. The pH of the solution was 5.2, and no catalyst was used in the synthesis method. The materials were slow dried, and grey precursors were obtained. The samples were then grounded using an agate mortar to obtain fine powders of ZnO, Zn_0.99_Cu_0.01_O and Zn_0.99_Mn_0.01_O. Thermal studies were done by using a simultaneous thermogravimetric analyzer (STA), SETARAM SETSYS Evolution 175. This equipment can give the thermogravimetric analysis (TGA) and differential scanning calorimetry (DSC) graphs simultaneously which is a more accurate way of determining the correct annealing temperature for producing single phase materials. Based on the TG/DSC, the precursors were annealed at 300 and 1200 °C for 24 h. Structural studies on the annealed samples were done by using X-ray diffraction (XRD), and the PANalytical X’pert Pro MPD diffractometer with a solid state detector (accelerator) was used. The XRD measurements were done using a Bragg-Brentano optical configuration in ambient conditions with the spinning mode to reduce effects of preferred orientation. The X-ray beam used was the CuK_α_ radiation. High-quality datasets were obtained with the highest peak around 10,000 counts as statistically required for proper quantitative XRD analysis [22–24]. Rietveld refinements of the XRD patterns for ZnO, Zn_0.99_Cu_0.01_O and Zn_0.99_Mn_0.01_O materials were done using the PanAlytical X’pert Highscore Plus software. For ZnO and Zn_0.99_Cu_0.01_O materials, the refinements were executed with the ICSD 67454 as the structural reference, while for refinements of Zn_0.99_Mn_0.01_O materials, ICSD 165003 is used as the structural reference. Both the structural reference has the hexagonal crystal structure with space group P63mc.

The morphology and crystallite size of the materials were examined by using field emission scanning electron microscopy (FESEM, JEOL JSM-7600F). The elemental analysis was examined using Oxford INCA X-MAC 51 XMX 0021 integrated with SEM. The light absorption properties of the materials were studied using the Perkin Elmer Lambda 950 UV-vis-NIR spectrophotometer. The measurements were done in reflection mode using the reduced reflectance technique in ambient conditions. The oxidation states, chemical environment and valence band studies of the materials were done by X-ray photoelectron spectroscopy (XPS) using the JEOL JPS-9200 equipment. The XPS spectra were recorded using a monochromator and Al K_α_ (1486 eV) radiation as the X-ray source. A charge neutralizer was used to reduce charging effects, and data were taken using a pass energy of 10 eV producing a full width at half maximum (FWHM) of 0.5 eV. The samples used were pelletized and, prior to insertion into the measuring chamber, were heated at 100 °C to minimize the presence of hydrocarbons on the surface of the materials.

## Results and Discussions

**Fig. 1:**
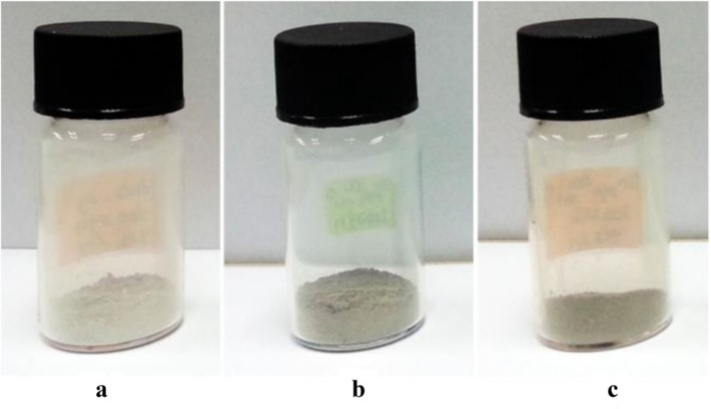
The colour of **a** ZnO, **b** Zn_0.99_Cu_0.01_O and **c** Zn_0.99_Mn_0.01_O material samples annealed at 300 °C

**Fig. 2:**
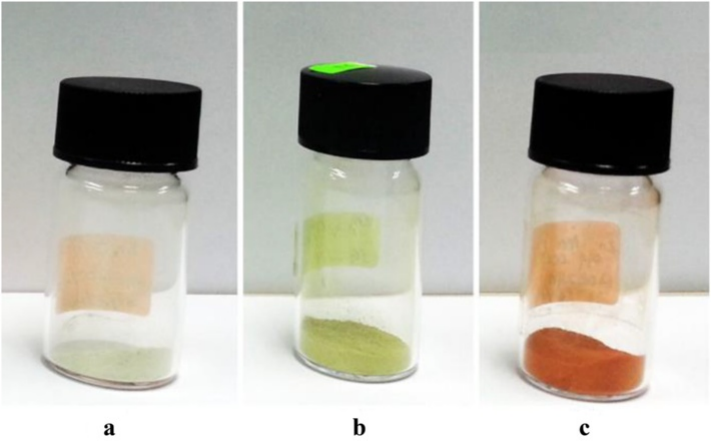
The colour of **a** ZnO, **b** Zn_0.99_Cu_0.01_O and **c** Zn_0.99_Mn_0.01_O material samples annealed at 1200 °C

The variations of colour for the comparison of undoped ZnO and doped materials (Zn_0.99_Cu_0.01_O and Zn_0.99_Mn_0.01_O) annealed at 300 and 1200 °C are shown in Figs. 1 and 2, respectively. At a lower annealing temperature of 300 °C, the colour for ZnO is light grey (Fig. 1a). For doped materials, Zn_0.99_Cu_0.01_O and Zn_0.99_Mn_0.01_O, the colours are a darker grey as shown in Fig. 1b, c, respectively. For the highest annealing temperature sample of 1200 °C, it is found that there is a big difference in colour for undoped ZnO and doped materials as can be seen in Fig. 2. The colour for ZnO, Zn_0.99_Cu_0.01_O and Zn_0.99_Mn_0.01_O samples are white, green and dark orange in colour, respectively. The difference in colour between undoped ZnO and doped ZnO materials gives clear indications of the different light absorption properties of the materials.

**Fig. 3:**
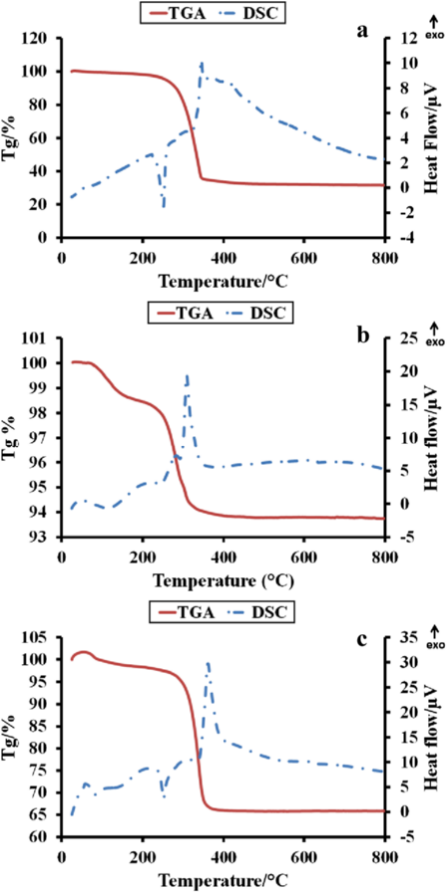
The STA result for **a** ZnO, **b** Zn_0.99_Cu_0.01_O and **c** Zn_0.99_Mn_0.01_O precursor

STA results are shown in Fig. 3. It can be observed that the formation temperature of the hexagonal structure for the Cu-doped sample is at a minimum (270 °C) compared to the pure ZnO (310 °C) and Mn-doped ZnO (300 °C). The annealing temperature is thus chosen as 300 and 1200 °C for the preparation of the nano and micron materials.

**Fig. 4:**
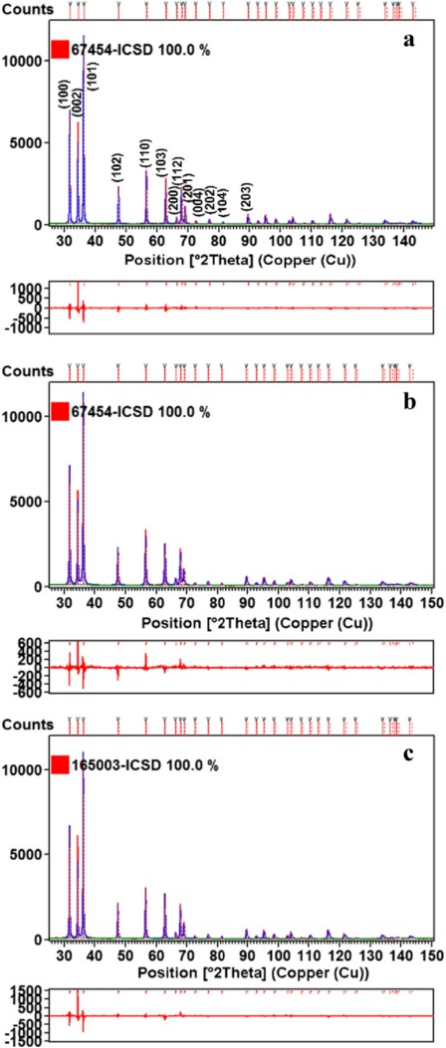
Rietveld refinements of XRD data of **a** ZnO, **b** Zn_0.99_Cu_0.01_O, and **c** Zn_0.99_Mn_0.01_O nanomaterials annealed at 300 °C

**Fig. 5:**
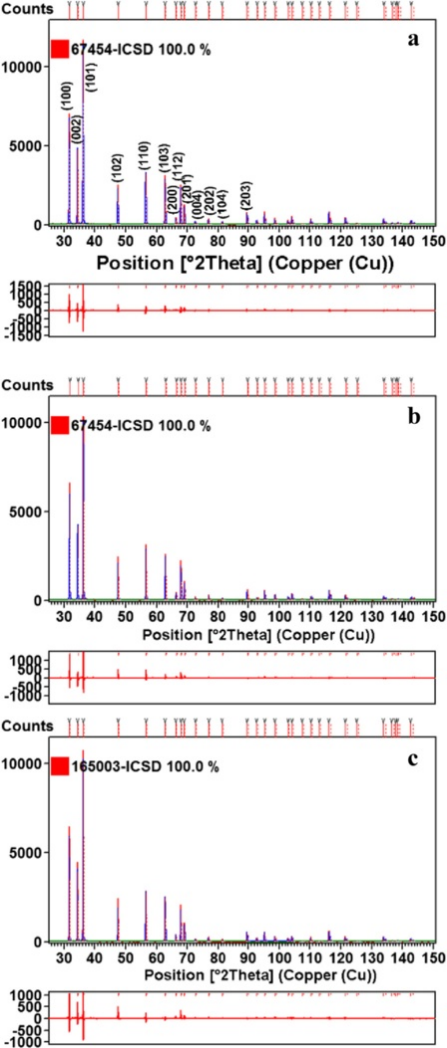
Rietveld refinements of XRD data of **a** ZnO, **b** Zn_0.99_Cu_0.01_O and **c** Zn_0.99_Mn_0.01_O nanomaterials annealed at 1200 °C

Phase studies for undoped ZnO, Zn_0.99_Cu_0.01_O and Zn_0.99_Mn_0.01_O samples are firstly carried out using XRD, and results show that all samples are phase pure. Quantitative analysis is later done via Rietveld refinement. Therefined XRD results for the undoped ZnO, Zn_0.99_Cu_0.01_O and Zn_0.99_Mn_0.01_O samples annealed at 300 and 1200 °C are shown in Figs. 4 and 5, respectively. Table 1 shows the crystallographic parameters extracted from the Rietveld refinements. It is found that the lattice parameters (a and c) of the samples annealed at 300 °C is larger than the lattice parameters of samples annealed at 1200 °C. Since the lower annealed samples of 300 °C results in nano-sized crystallites whereas the 1200 °C gives micron-sized crystallites, this shows that there is lattice expansion in ZnO nanomaterials. This phenomenon of lattice expansion in nanostructured materials can be explained by the fewer number of atoms in the nanocrystallites compared to the micron-sized particles which results in fewer inter-atomic interactions between the atoms in the lattice resulting in a decrease of the electrostatic coulombic force of attraction. Thus, the inter-planar d-spacings are larger. The *c*/*a* values of the materials are also larger for the nanomaterials than the very crystalline materials indicating that the atomic packing of the atoms is denser for the conventional materials with large micron crystal size. It is observed that the oxygen site occupancy factors are both full for the nano- and micron-sized materials of ZnO showing that Zn has a very strong affinity to oxygen and that is why they are very stable oxides. For the doped compounds, there is a small amount of oxygen vacancy for the Cu-doped materials and the presence of O vacancy only exists in the nanomaterial of Mn-doped samples. These findings indicate that there are small amounts of vacancies present in the Cu- and Mn-doped ZnO but not for the pure ZnO. Therefore, results show that there is no oxygen vacancies in nano or micron ZnO as is speculated by several researchers [16, 17].

**Table 1:** Crystallographic parameters of all samples extracted from the Rietveld refinements of the XRD datasets

Samples	Temperature (°C)	*a* = *b* (Å)	*c* (Å)	*V* (Å^3^)	*c*/*a*	Rw	*χ* ^2^	s.o.f. Zn	s.o.f. Cu	s.o.f. Mn	s.o.f. of O
ZnO	300	3.2497	5.2063	47.6153	1.6021	9.3	1.5	0.9601	–	–	1.0000
	1200	3.2496	5.2032	47.5828	1.6012	12.3	1.9	0.9739	–	–	1.0000
Zn_0.99_Cu_0.01_O	300	3.2496	5.2067	47.6153	1.6023	7.6	1.3	0.9372	0.0093	–	0.9995
	1200	3.2502	5.2050	47.6170	1.6014	13.9	1.8	0.9631	0.0082	–	0.9983
Zn_0.99_Mn_0.01_O	300	3.2499	5.2061	47.6182	1.6019	9.7	1.7	0.9075	–	0.0092	0.9995
	1200	3.2517	5.2063	47.6743	1.6011	14.6	1.8	0.9606	–	0.0055	1.0000

*V* volume, *RW* weighted R profile, *χ* ^*2*^ goodness of fit, *s.o.f.* site occupancy factor

**Fig. 6:**
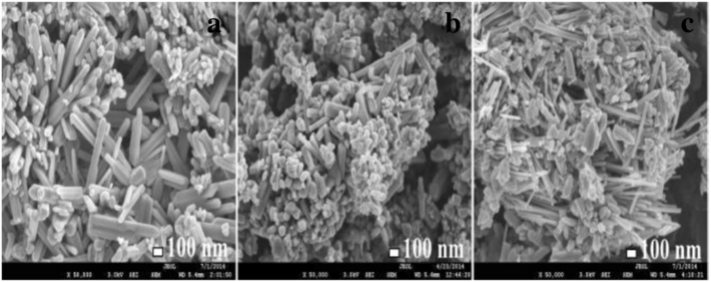
SEM images of **a** ZnO, **b** Zn_0.99_Cu_0.01_O and **c** Zn_0.99_Mn_0.01_O nanostructures annealed at 300 °C

**Fig. 7:**
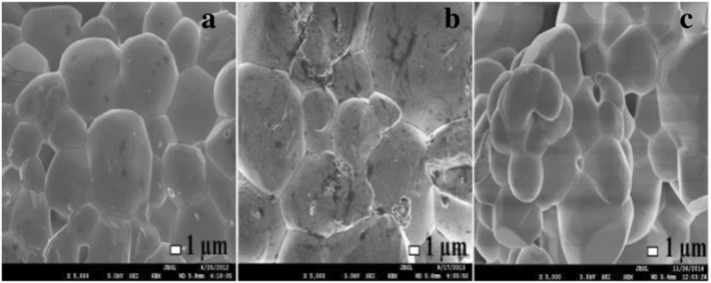
SEM images of **a** ZnO, **b** Zn_0.99_Cu_0.01_O and **c** Zn_0.99_Mn_0.01_O nanostructures annealed at 1200 °C

The morphologies for ZnO, Zn_0.99_Cu_0.01_O and Zn_0.99_Mn_0.01_O nanostructures annealed at 300 °C are shown in Fig. 6. The summary of the morphology and crystallite dimensions (length and diameter) for the materials are given in Table 2. It can be seen that the morphology for undoped ZnO and doped materials consist of a mixture of long nanorods and small amounts of tiny spherical shapes. It is also found that Zn_0.99_Mn_0.01_O nanorods has the smallest diameter with an average of 50.16 nm, followed by Zn_0.99_Cu_0.01_O and ZnO nanostructures with average diameters of 69.38 and 69.4 nm, respectively. Figure 7 shows the morphologies for ZnO, Zn_0.99_Cu_0.01_O and Zn_0.99_Mn_0.01_O annealed at 1200 °C. It can be observed that the morphology for undoped ZnO and doped materials consist of spherical-like shapes. It is found that the Zn_0.99_Cu_0.01_O samples have the largest crystallite size followed by ZnO and Zn_0.99_Mn_0.01_O samples. The difference in crystallite size is believed to be related to the crystal growth rate of the materials because all of them are annealed at the same annealing temperature with the same time duration. In this case, Zn_0.99_Cu_0.01_O samples have a higher crystal growth rate resulting in larger crystallites after the annealing process. The dopant content is confirmed via SEM EDX, and the values obtained are very close to the synthesized values as listed in Table 3.

**Table 2:** The morphology, dimension (diameter and length), valence band maximum (VBM) and band gap energy (*E*_g_) of ZnO, Zn_0.99_Cu_0.01_O and Zn_0.99_Mn_0.01_O samples annealed at 300 °C and 1200 °C for 24 h

Samples	Temperature (°C)	Morphology	Average diameter (nm)	Average length (nm)	*E*_g_ (eV) (±0.01)	VBM (eV) (FWHM = 0.5 eV)
ZnO	300	Mixture of long nanorods and spherical shapes	69.40	427.00	3.33	2.4
	1200	Spherical shapes	4706.55	NA	3.19	1.9
Zn_0.99_Cu_0.01_O	300	Mixture of long nanorods and spherical shapes	69.38	342.01	3.29	2.5
	1200	Spherical shapes	8027.82	NA	2.98	1.9
Zn_0.99_Mn_0.01_O	300	Mixture of long nanorods and spherical shapes	50.16	584.10	3.32	2.4
	1200	Spherical shapes	4419.95	NA	2.44	2.6

*NA* not applicable

**Table 3:** Energy dispersive X-ray (EDX) quantitative analysis of Zn_0.99_Cu_0.01_O and Zn_0.99_Mn_0.01_O samples

Samples	Temperature (°C)	Element	Atom (%)	Calculated stoichiometry from EDX	Calculated stoichiometry from experiment
Zn_0.99_Cu_0.01_O	300	Cu K	0.93	0.0093	0.01
		Zn K	99.07	0.9907	0.99
	1200	Cu K	0.96	0.0096	0.01
		Zn K	99.04	0.9904	0.99
Zn_0.99_Mn_0.01_O	300	Mn K	0.96	0.0096	0.01
	1200	Zn K	99.04	0.9904	0.99
		Mn K	0.99	0.0099	0.01
		Zn K	99.01	0.9901	0.99

**Fig. 8:**
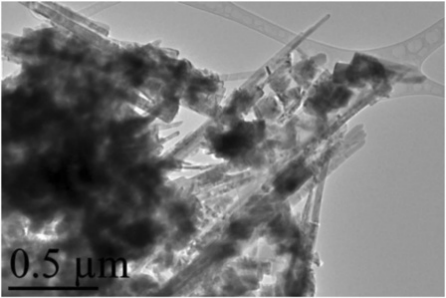
TEM results for ZnO nanorods annealed at 300 °C

**Fig. 9:**
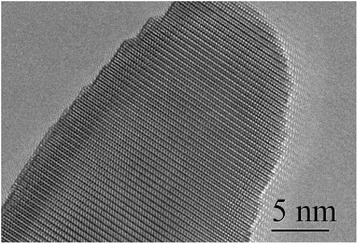
HRTEM results for ZnO nanorods annealed at 300 °C

**Fig. 10:**
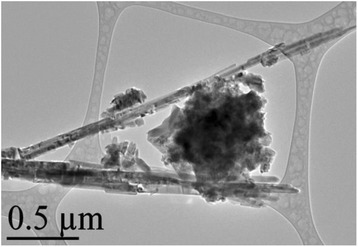
TEM results for Zn_0.99_Cu_0.01_O nanorods annealed at 300 °C

**Fig. 11:**
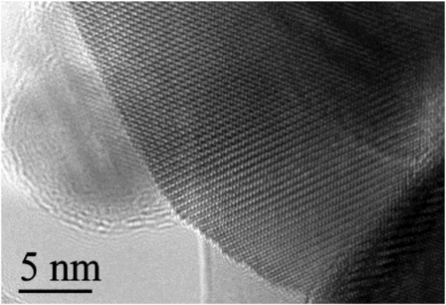
HRTEM results for Zn_0.99_Cu_0.01_O nanorods annealed at 300 °C

**Fig. 12:**
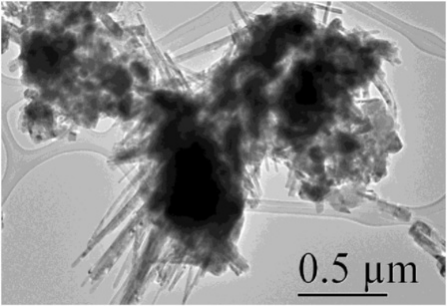
TEM results for Zn_0.99_Mn_0.01_O nanorods annealed at 300 °C

**Fig. 13:**
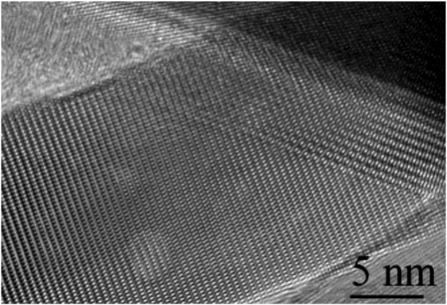
HRTEM results for Zn_0.99_Mn_0.01_O nanorods annealed at 300 °C

The in-depth studies of the morphologies for ZnO, Zn_0.99_Cu_0.01_O and Zn_0.99_Mn_0.01_O nanostructures annealed at 300 °C are investigated using HRTEM. Lower and higher magnifications for ZnO, Zn_0.99_Cu_0.01_O and Zn_0.99_Mn_0.01_O nanostructures are shown in Figs. 8, 9, 10, 11, 12, and 13, respectively, to illustrate the morphology and size of the materials and to show the nanocrystalline nature of the structures. The higher resolution TEM images confirm the nanorod shapes of the crystallites but the seemingly spherical shapes shown in the SEM are actually polyhedral type crystallites with angular crystal faces. Thus, TEM has the ability to reveal the actual morphology of the nanocrystallites to be actually faceted polyhedral crystals with smooth faces. The nanorods are also single crystals as can be clearly seen in the HRTEM images showing the regular arrangement of the lattice of the ZnO, Zn_0.99_Cu_0.01_O and Zn_0.99_Mn_0.01_O materials.

**Fig. 14:**
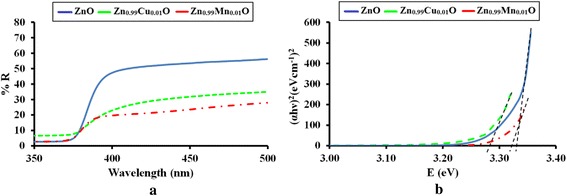
The results of **a** UV-visible spectra and **b** Tauc plots for ZnO, Zn_0.99_Cu_0.01_O and Zn_0.99_Mn_0.01_O samples annealed at 300 °C

**Fig. 15:**
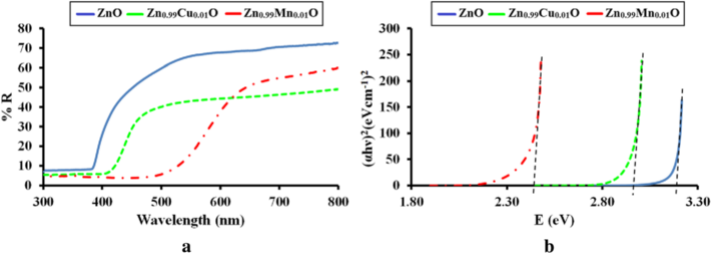
The results of **a** UV-visible reflectance spectra and **b** Tauc plots for ZnO, Zn_0.99_Cu_0.01_O and Zn_0.99_Mn_0.01_O samples annealed at 1200 °C

The optical band gap properties of the ZnO, Zn_0.99_Cu_0.01_O and Zn_0.99_Mn_0.01_O samples are studied by UV-visible spectroscopy. The results for ZnO, Zn_0.99_Cu_0.01_O and Zn_0.99_Mn_0.01_O samples annealed at 300 and 1200 °C are shown in Figs. 14a and 15a, respectively. It is observed that the absorption edges of the doped materials (Zn_0.99_Cu_0.01_O and Zn_0.99_Mn_0.01_O) are shifted to the right compared to undoped ZnO. These shifts towards the higher energy wavelength of light imply band gap narrowing in the doped compounds as confirmed by the analysis of the spectroscopic data via Tauc plots. From the UV-visible results, Tauc plots are drawn by using the Tauc equation below

1 $$ {\left(\alpha h\upsilon \right)}^2=C^{\prime}\left(h\upsilon -{E}_{\mathrm{g}}\right) $$

where *α* is the absorption coefficient of the material, *λ* is the wavelength, *h* is the Planck’s constant, *C*′ is the proportionality constant, *υ* is the frequency of light and *E*_g_ is the band gap energy. The graph plotted is (*αhυ*)^2^ vs. *hυ* and from Eq. (1) extrapolating the linear part of the graph until it meets the *x*-axis will give the value of the band gap [8]. The Tauc plots for the ZnO, Zn_0.99_Cu_0.01_O and Zn_0.99_Mn_0.01_O samples annealed at 300 and 1200 °C are shown in Figs. 14b and 15b, respectively. The band gap values obtained from the plots are listed in Table 2. The dimensions of the crystallites are also listed so that the effect of crystal dimensions and band gaps can be easily seen. At 300 °C, the band gap energies for ZnO, Zn_0.99_Cu_0.01_O and Zn_0.99_Mn_0.01_O samples are 3.33, 3.288 and 3.32 eV, respectively. The Zn_0.99_Cu_0.01_O sample has the smallest band gap compared to ZnO and Zn_0.99_Mn_0.01_O, and this is due to the influence of the Cu content because CuO has a smaller band gap of between 1.2 and 1.3 eV [25]. It is observed that for all groups of samples, the band gap energy of the nanomaterials is larger than their micron-sized materials. Band gap widening of nanomaterials are attributed to the quantum mechanical effects of the low-dimensional crystallites. At these length scales, overlapping energy levels spread out to become more quantized producing band gap widening in the materials. It is also observed that doped materials have the opposite behaviour, that is, exhibiting band gap narrowing with respect to the undoped samples for each temperature. The band gap energies of doped compounds (Zn_0.99_Cu_0.01_O and Zn_0.99_Mn_0.01_O) are smaller than undoped ZnO, and this is true for all cases whether the crystallites are nano or micron in size. At the annealing temperature of 1200 °C (micron samples), the band gap energies for ZnO, Zn_0.99_Cu_0.01_O and Zn_0.99_Mn_0.01_O samples are 3.19, 2.98 and 2.44 eV, respectively. At this temperature, Zn_0.99_Mn_0.01_O sample has the smallest band gap compared to ZnO and Zn_0.99_Cu_0.01_O samples. This is attributed to the presence of a mixture of Mn^2+^, Mn^3+^ and Mn^4+^ in the Zn_0`.99_Mn_0.01_O lattice because Mn can exist in different oxidation states. The presence of the Mn^3+^ and Mn^4+^ produces colour centres due to unpaired electrons in the d orbital that narrows the band gap which is explained in detail in the XPS discussion below.

**Fig. 16:**
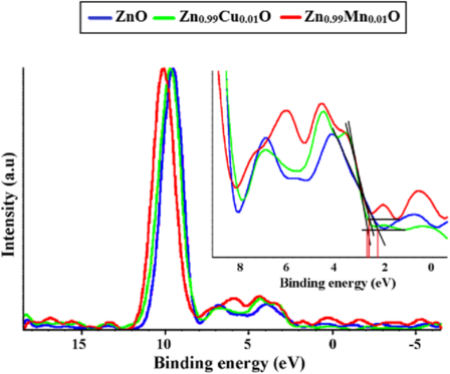
The comparison of XPS valence band for ZnO, Zn_0.99_Cu_0.01_O and Zn_0.99_Mn_0.01_O nanomaterials annealed at 300 °C

**Fig. 17:**
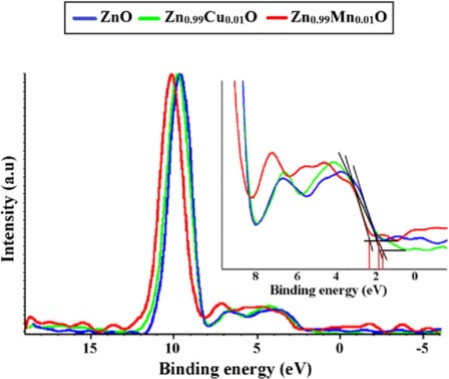
The comparison of XPS valence band for ZnO, Zn_0.99_Cu_0.01_O and Zn_0.99_Mn_0.01_O micron materials annealed at 1200 °C

**Fig. 18:**
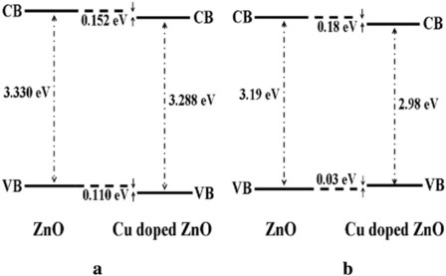
The energy level diagram of undoped ZnO and Cu-doped ZnO of **a** nano and **b** micron samples

**Fig. 19:**
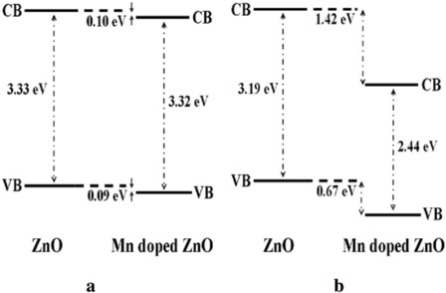
The energy level diagram of undoped ZnO and Mn-doped ZnO of **a** nano and **b** micron samples

Further studies of the valence band for ZnO, Zn_0.99_Cu_0.01_O and Zn_0.99_Mn_0.01_O samples annealed at 300 and 1200 °C and their relation to the band gap energies are carried out via XPS. The XPS valence band spectra for Zn_0.99_Cu_0.01_O and Zn_0.99_Mn_0.01_O samples annealed at 300 and 1200 °C are shown in Figs. 16 and 17 with respect to the valence band of the pure undoped ZnO. The valence band maximum for all samples is listed in Table 2. At 300 °C, it is found that there is a valence band shift towards the higher binding energy side of the energy axis for the doped nanomaterials (Zn_0.99_Cu_0.01_O and Zn_0.99_Mn_0.01_O) with respect to ZnO (insets are magnified parts of these regions to show the shifts more clearly). This suggests that for Zn_0.99_Cu_0.01_O and Zn_0.99_Mn_0.01_O, the valence band maximum shifts downwards with respect to the original pure ZnO. The energy levels (to scale) of nanostructures for the Zn_0.99_Cu_0.01_O and Zn_0.99_Mn_0.01_O materials (with respect to ZnO nanostructures) are constructed and shown in Figs. 18a and 19a, respectively, with the Fermi level at zero point. The valence band of the doped nanomaterials is shifted downwards with respect to the pure ZnO nanomaterial, and the lowest unoccupied molecular orbital (LUMO) of the conduction band of the Zn_0.99_Cu_0.01_O and Zn_0.99_Mn_0.01_O nanostructures also shifts downwards with respect to the LUMO of the ZnO material. Therefore, this shows that for Zn_0.99_Cu_0.01_O and Zn_0.99_Mn_0.01_O nanostructures, not only does the valence band shift downwards but the conduction band also shifts downwards with respect to the energy levels of the pure ZnO nanomaterials but to different degrees. The narrowing of the band gaps of the doped nanomaterials can then be said to be mainly due to the downward shifts of the LUMOs of the conduction bands because the shifts occur at larger values than the shifts of the HOMO. In other words, the narrowing of the band gaps in the doped nanomaterials can be mainly attributed to the downward shift of the conduction bands.

**Fig. 20:**
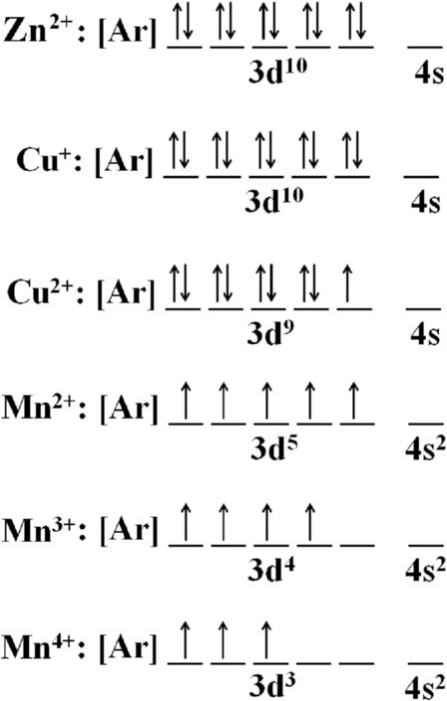
The electronic configuration for oxidation states present in Zn_0.99_Cu_0.01_O and Zn_0.99_Mn_0.01_O materials

It is also observed that there are shifts in the 3d orbital at around 10 eV. For both cases of nano- and micron-sized materials, the shift is the same, that is, there is a slight left shift for the Cu-doped material and a larger left shift for the Mn-doped material. This can be explained by the electronic configurations of the transition metals. Since Cu exists in Cu^+^ and Cu^2+^, there is only one electron missing in the Cu^2+^ ion (Fig. 20). Thus, this is not such a great difference compared to the Zn^2+^ electronic configuration which is the same as the Cu^+^ case. Therefore, the left shift for the Cu-doped material is very slight. In case of the Mn-doped samples, the situation is very different because Mn^2+^, Mn^3+^ and Mn^4+^ have vast differences in their electronic configuration compared to Zn^2+^. As can be seen from Fig. 20, the electrons missing from the full 3d orbital of Mn ions range from five to seven electrons in their orbitals depending on the oxidation states. This accounts for the large left shift observed for the Mn-doped samples with respect to the ZnO 3d peak.

**Fig. 21:**
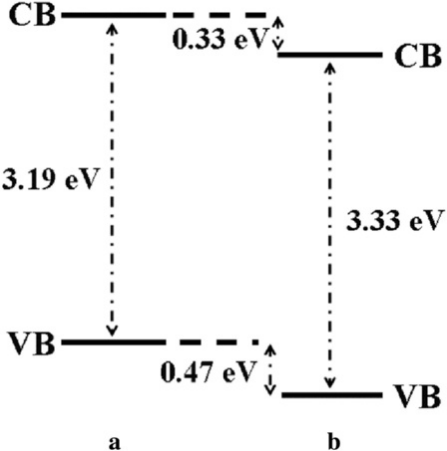
The energy level diagram for ZnO material of **a** micron and **b** nanomaterials

**Fig. 22:**
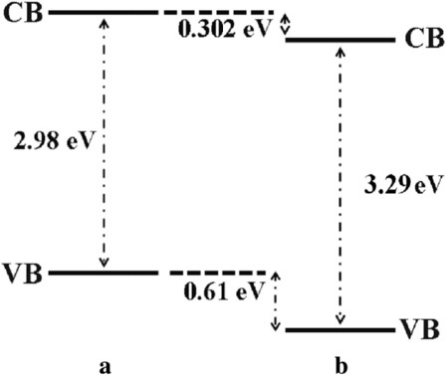
The energy level diagram for Zn_0.99_Cu_0.01_O material of **a** micron and **b** nanomaterials

**Fig. 23:**
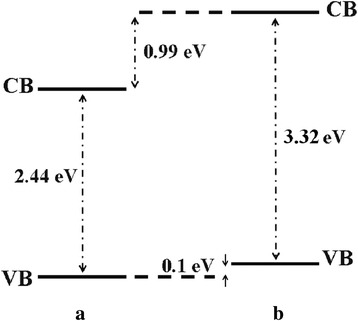
The energy level diagram for Zn_0.99_Mn_0.01_O material of **a** micron and **b** nanomaterials

At 1200 °C, that is, for the micron-sized materials, it is found that there is a valence band shift towards the lower binding energy side of the energy axis for the Zn_0.99_Cu_0.01_O material with respect to the ZnO material. This suggests that for Zn_0.99_Cu_0.01_O micron material, the valence band maximum shifts upwards towards the conduction band. Similarly, analyzing the situation by constructing the energy levels as shown in Fig. 18b, it is found that the LUMO of the conduction band is conversely shifted downwards. Therefore, the narrowing of the band gap of the micron-sized Zn_0.99_Cu_0.01_O material is due to both the upward and downward shifts of the valence band and conduction band, respectively. It is observed that the band gap narrowing in the micron material is higher with a 0.21 eV difference compared to only 0.04 eV difference for the nanomaterial. In the case of the Mn-doped micron-sized material, the HOMO and LUMO is shifted downwards with respect to the HOMO and LUMO of the ZnO material (Fig. 19b). Band gap narrowing for the Mn-doped micron material is affected mostly by the downward shift of the HOMO of the conduction band of the material. Therefore, the narrowing of the band gap in the case of the Mn-doped micron-sized material is attributable to the downward shift of the conduction band. It is also observed that the degree of shifts of the conduction and valence bands is larger and very much more obvious in the micron Mn-doped material compared to the nano Mn-doped material. The band gap narrows by 0.75 eV for the micron material compared to only 0.01 eV for the nanomaterial. To see the results of the shifts of the nanomaterials with respect to the micron state, the energy level diagrams for all materials are shown in Figs. 21, 22 and 23 for the ZnO, Zn_0.99_Cu_0.01_O and Zn_0.99_Mn_0.01_O samples, respectively.

**Fig. 24:**
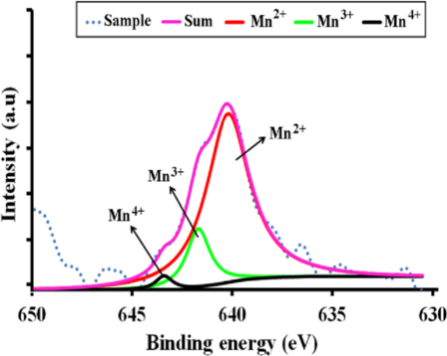
The deconvolution of Mn 2p_3/2_ of Zn_0.99_Mn_0.01_O samples annealed at 300 °C

**Fig. 25:**
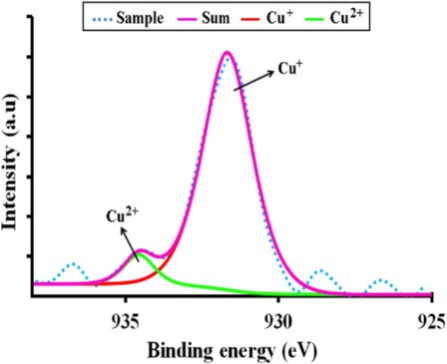
The deconvolution of Cu 2p_3/2_ of Zn_0.99_Cu_0.01_O samples annealed at 300 °C

The doped transition metals seem to exist in more than one oxidation states as obtained from XPS core level peaks for Mn 2p_3/2_ and Cu 2p_3/2_ shown in Figs. 24 and 25, respectively. Deconvoluting the peaks, quantitative amounts of the different oxidation states are obtained as shown in Table 4. The existence of the transition metals in different oxidation states especially Mn accounts for the left shifts of the 3d peaks of the XPS results. The large left shift for the Mn-doped sample is due to the quite considerable electrons missing from the orbitals as compared to the Zn^2+^ ions which have all the orbitals full. For the Cu-doped sample, since there is only one electron missing in the orbital for the Cu^2+^ ion, the shift is very slight.

**Table 4:** The percentage of oxidation states of substituent elements (Cu and Mn) in ZnO samples obtained through XPS quantitative studies

Samples	Temperature (°C)	Oxidation rates (%)				
		Cu^+^	Cu^2+^	Mn^2+^	Mn^3+^	Mn^4+^
Zn_0.99_Cu_0.01_O	300	92.9	7.1	–	–	–
	1200	13.11	86.89	–	–	–
Zn_0.99_Mn_0.01_O	300	–	–	82.0	15.4	2.6
	1200	–	–	87.6	8.2	4.2

Results have shown that the mechanism for band gap narrowing of doped samples is different in the nano and micron cases. Crystallinity of the samples thus plays a very important part in the band gap change of materials. It has also been demonstrated here that it is possible to study the conduction and valence band shifts of materials by employing the powerful XPS and UV-visible spectroscopic methods together.

## Conclusions

This work has shown that band gap widening occurs in all nanostructured ZnO and doped ZnO with respect to the micron materials. However, doping ZnO with the transition metals Cu and Mn causes band gap narrowing in both the nano and micron materials. The mechanism of the band gap widening in the nanostructured materials is very complex. For ZnO nanostructured material, the band gap widening can be mainly attributed to the larger downward shift of the VB (Fig. 21). For the Cu-doped ZnO, the band gap widening is due to the larger downward shift of the valence band (Fig. 22). For the Mn-doped ZnO, the band gap widening is mainly due to the larger upward shift of the conduction band (Fig. 23). Band gap narrowing of doped compounds with respect to pure ZnO can be said to be mainly due to the downward shifts of the conduction band (Fig. 18) for both the nanostructured and micron materials. Thus, mechanisms for band gap narrowing and widening in materials are very complex processes depending on whether they are nano- or micron-sized crystallites and the type of elements involved in the doping process.

## References

[1] Willander M, Zhao QX, Hu QH, Klason P, Kuzmin V, Al-Hilli SM (2008). Fundamentals and properties of zinc oxide nanostructures: optical and sensing applications. Supperlattice Microst..

[2] Caglar M, Ilican S, Caglar Y, Yakuphonoglu F (2009). Electrical and optical properties of ZnO nanostructured thin film. Mater. Chem. Phys..

[3] Sahoo T, Tripathy SK, Yu YT, Ahn HK, Shin DC, Lee IH (2008). Morphology and crystal quality investigation of hydrothermally synthesized ZnO micro rods. Mater. Res. Bull..

[4] Mazloumi M, Taghavi S, Arami H, Zanganeh S, Kajbafla A, Shayegh MR (2009). Self assembly of ZnO nanoparticles and subsequent formation of hollow microspheres. J. Alloys and Comp..

[5] Li F, Bi W, Liu L, Li Z, Huang X (2009). Preparation and characterization of ZnO nanospindles and ZnO @ ZnS core-shell microspindles. Colloids and Surfaces A: Physiocochem. Eng. Aspects..

[6] Tong Y, Cheng J, Liu Y, Siu GG (2009). Enhanced photocatalytic performance of ZnO hierarchical nanostructures synthesized via a two-temperature aqueous solution route. Scripta. Mat..

[7] Hong RY, Li JH, Chen LL, Liu DQ, Li HZ, Zheng Y (2009). Synthesis, surface modification and photocatalytic property of ZnO nanoparticles. Powder Technol..

[8] Zhang H, Chen B, Jiang H, Wang C, Wang H, Wang X (2011). A strategy for ZnO nanorod mediated multi-mode cancer treatment. Biomaterials.

[9] Rusdi R, Rahman AA, Mohamed NS, Kamarudin N, Kamarulzaman N (2011). Preparation and band gap energies of ZnO nanotubes, nanorods and spherical nanostructures. Powder Technol..

[10] Caglar M, Caglar Y, Aksoy S, Ilican S (2010). Temperature dependence of d optical band gap and electrical conductivity of sol–gel derived undoped and Li-doped ZnO films. Appl. Surf. Sci..

[11] Shan FK, Liu GX, Lee WJ, Shin BC (2006). Stokes shift, blue shift and red shift of ZnO-based thin films deposited by pulsed-laser deposition. J. Cryst. Growth.

[12] Shan FK, Yu YS (2004). Band gap energy of pure and Al-doped ZnO thin films. J. Eur. Ceram. Soc..

[13] Salleh R, Prakoso SP, Fishli A (2012). The influence of Fe doping on the structural, magnetic and optical properties of nanocrystalline ZnO particles. J. Magn. Magn. Mater..

[14] Chen Y, Xu XL, Zhang GH, Xue H, Ma SY (2010). Blue shift of optical band gap in Er-doped ZnO thin films deposited by direct current reactive magnetron sputtering technique. Physica E.

[15] Liu H, Yang J, Hua Z, Zhang Y, Yang L, Xiao L (2010). The structure and magnetic properties of Cu-doped ZnO prepared by sol–gel method. Appl. Surf. Sci..

[16] Ansari SA, Khan MM, Kalathil S, Nisar A, Lee J, Cho MH (2013). Oxygen vacancy induced band gap narrowing of ZnO nanostructures by an electrochemically active biofilm. Nanoscale.

[17] Wang J, Wang Z, Huang B, Ma Y, Liu Y, Qin X (2012). Oxygen vacancy induced band-gap narrowing and enhanced visible light photocatalytic activity of ZnO. ACS Appl. Mater. Interfaces.

[18] Bitenc M, Orel ZC (2009). Synthesis and characterization of crystalline hexagonal bipods of zinc oxide. Mater. Res. Bull..

[19] Cao Z, Zhang Z, Wang F, Wang G (2009). Synthesis and UV shielding properties of zinc oxide ultra fine particles modified with silica and trimethyl soloxane. Colloids Surface A.

[20] Lee YC, Hu SY, Water W, Toing KK, Feng ZC, Chen YT (2009). Rapid thermal annealing effects on the structural, and optical properties of ZnO films deposited on Si substrates. J Luminescence.

[21] Henseler MJH, Lee WCT, Miller P, Durbin SM, Reevers RJ (2006). Optical and photoelectrical properties of ZnO thin films and the effect of annealing. J. Cryst. Growth.

[22] David WIF, Shankland K, McCusker LB, Baerlocher C (2002). Structure determination from powder diffraction data.

[23] Zenkins R, Snyder RL (1996). Introduction to X-ray powder diffractometry.

[24] Pecharsky VK, Zavalij PY (2005). Fundamentals of powder diffraction and structural characterization of materials.

[25] Johan MR, Suan MSM, Hawari NL, Ching HA (2011). Annealing effects on the properties of copper oxide thin films prepared by chemical deposition. Int. J. Electrochem. Sci..

